# Assessment of work-integrated learning: comparison of the usage of a grading rubric by supervising radiographers and teachers

**DOI:** 10.1002/jmrs.39

**Published:** 2013-12-25

**Authors:** Andrew J Kilgour, Peter W Kilgour, Tania Gerzina, Beverly Christian

**Affiliations:** 1 Charles Sturt UniversityWagga Wagga, NSW, Australia; 2 Avondale College of Higher EducationCooranbong, NSW, Australia; 3 Dental Educational Research, Faculty of Dentistry, Jaw Function and Orofacial Pain Research Unit, Westmead Centre for Oral Health, C24- Westmead Hospital, The University of SydneySydney, NSW, 2006, Australia

**Keywords:** Assessment, pre-service teachers, radiography, rubric, work-integrated learning

## Abstract

**Introduction**Professional work-integrated learning (WIL) that integrates the academic experience with off-campus professional experience placements is an integral part of many tertiary courses. Issues with the reliability and validity of assessment grades in these placements suggest that there is a need to strengthen the level of academic rigour of placements in these programmes. This study aims to compare the attitudes to the usage of assessment rubrics of radiographers supervising medical imaging students and teachers supervising pre-service teachers.

**Methods**WIL placement assessment practices in two programmes, pre-service teacher training (Avondale College of Higher Education, NSW) and medical diagnostic radiography (Faculty of Health Sciences, University of Sydney, NSW), were compared with a view to comparing assessment strategies across these two different educational domains. Educators (course coordinators) responsible for teaching professional development placements of teacher trainees and diagnostic radiography students developed a standards-based grading rubric designed to guide assessment of students’ work during WIL placement by assessors. After ∼12 months of implementation of the rubrics, assessors’ reaction to the effectiveness and usefulness of the grading rubric was determined using a specially created survey form. Data were collected over the period from March to June 2011. Quantitative and qualitative data found that assessors in both programmes considered the grading rubric to be a vital tool in the assessment process, though teacher supervisors were more positive about the benefits of its use than the radiographer supervisors.

**Results**Benefits of the grading rubric included accuracy and consistency of grading, ability to identify specific areas of desired development and facilitation of the provision of supervisor feedback. The use of assessment grading rubrics is of benefit to assessors in WIL placements from two very different teaching programmes.

**Conclusion**Radiographers appear to need more training in the rubric's use, whereas teachers are found to generally use it appropriately. There are implications drawn from this finding that are applicable to health science and medical education in general.

## Introduction

There is a recognised need to strengthen the academic rigour of professional work-integrated learning (WIL) placement assessment.^1^ Planning, administering, grading and evaluating assessment of students in the university campus setting can be a complex task with quality of a student's performance having multiple informing criteria, some of which may be quite abstract.^2^ When the learning setting is a WIL placement, additional factors further complicate the validity and reliability of the assessment process. These factors include having a large number of assessors whose interpretations of assessment requirements may be different; the variability between WIL placement environments; the fact that many assessors supervise students in addition to a full-time workload and many other variables, which simply are not present in the on-campus environment. Orrell et al.^3^ state that the assessment of student performance and workplace practice is the most complex of all assessment modes.

It is common that university educators and course coordinators have limited ability to monitor teaching practices of volunteer supervisors who function as assessors in the workplace on a regular basis. The provision of assessment guidelines utilising a grading rubric with descriptors of the professional standards and qualities, and training in the application of these standards, is a constructive way of engaging off-campus WIL supervisors in the implementation of defensible, valid assessment.^3^

The aim of this study was to compare the attitudes of teachers and health professionals (in this case radiographers) who supervise undergraduate students in the workplace about the use of a purpose designed assessment rubric that has common structural elements and criteria. By default, teachers have training in the principles and practices of education, which includes assessment of a student's performance. In contrast, health professionals have no such training, unless they have elected to undertake it at a postgraduate level. This study investigates whether this educational background is a contributing factor in attitudinal differences between teachers and health professionals towards WIL assessment. It also draws conclusions as to whether educational training may be the key to improving the reliability and validity of WIL assessment practices of health professional supervisors.

## Method

This study was granted approval by the Human Research Ethics Committee of Avondale College of Higher Education.

### Sample

Data collection was over the period from March to June 2011. Participants were recruited by a letter sent to all schools and radiology departments accepting students from the institutions involved in the study. The letters were addressed to the primary student supervision contact in each institution who was asked to invite all staff involved in student supervision to participate. Completed surveys were received from radiographers employed in public and private radiology departments predominantly in metropolitan and regional New South Wales (NSW), with a small number of interstate radiographers also responding. Avondale College sends pre-service teachers to public and independent schools in all Australian states and territories, and both islands of New Zealand. Completed surveys were received from a broad cross-section of these schools.

### Grading rubric

**Figure 1:**
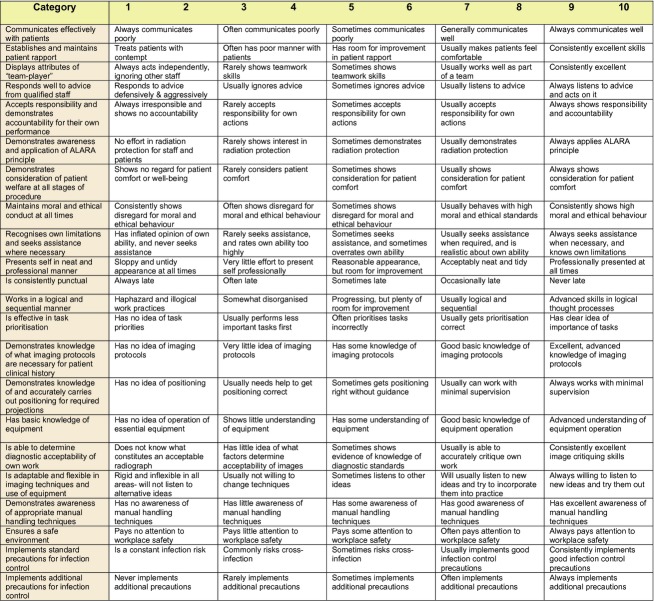
Radiographer's rubric.

A grading rubric was developed for the use of radiography clinical supervisors that plotted assessment categories on the vertical axis, against achievement standards for each of these categories on the horizontal axis (See Fig. 1).

This rubric was then adapted for use by teacher supervisors. Although the assessment categories were adapted for the two different professions, the principles by which they function in the assessment of WIL for undergraduate students are the same. This was not deemed a methodological problem, as the study compared the attitudes of supervisors in the two professions to the use of a suitable rubric. It was not a study of the rubric itself.

### Survey instrument

A cross-sectional survey instrument^4^ was specially constructed to determine how workplace supervisors used the rubric provided and their opinions on its ease of use, its accuracy and its effectiveness. This instrument was piloted with selected radiography and teaching supervisors, and modified for reliability and validity based on their comments. The final instrument consisted of two demographic questions, two questions asking the level of familiarity with the appropriate rubric, five Likert scale questions with five options (ranging from ‘strongly agree’ to ‘strongly disagree,’ with ‘not applicable’ as the final option), which asked about perceived effectiveness of the rubric for WIL assessment, and two short answer questions which allowed for further comments on best and worst aspects of rubric use and any suggested improvements in its design and usability. The survey instrument was distributed and returned in hard copy by conventional mail.

The survey instrument face-validity was ascertained by iterative consultation with both professional and non-professional radiography and teacher academics. Any comments on review of the survey instruments were absorbed into the content.

### Data analysis

Quantitative data were analysed using Graphpad Prism software (version 5.0, La Jolla, CA, USA), with a non-parametric test (Mann–Whitney)^5^ used to compare the median response to each question. These medians were compared for significant differences between the two professional groups represented. All quantitative data analysis was verified by a senior researcher with statistical experience.

Qualitative data from the survey were aligned with informal comments received by the authors and assisted in organising responses in a thematic analysis. This was done using NVivo 9™ (Doncaster, Victoria, Australia) qualitative data analysis software,^6^ using a phenomenonological approach.^4^ Construct and content validity^7^ was determined by submitting the survey to review by members of both professions. Alternate-form reliability^8^,^9^ was determined by using two questions of identical meaning but changed wording.

## Results and Discussion

### Quantitative

One hundred responses were received from radiographer supervisors and 112 responses from teacher supervisors. This represented response rates of 30% of the total number sent out for the pre-teacher group and 55% of the total number sent out for the diagnostic radiography group.

After the teaching supervisors and the medical imaging supervisors were surveyed, initial analysis provided findings on the following:

The practice experience of the supervisors;
The qualifications of the supervisors;
The supervisors’ familiarity with the grading rubric; and
The supervisors’ use of the grading rubric.

#### Closed ended questions

Respondents were asked to indicate a range that their years of practice fell into, and the modal range of respondents in both groups was 6–10 years (radiographers SD = 1.422, teachers SD = 1.231), with a range from less than 5 years to greater than 30 years in both professional groups. This renders the professional groups comparable in terms of experience.

In terms of qualifications, radiographers ranged from those with their highest qualification at certificate/associate diploma/diploma level, to those who had a Masters by research. Teachers ranged from a certificate/associate diploma/diploma qualification, to those who had a Masters by coursework. The modal level of qualification for both groups was a bachelor degree.

Respondents were asked to answer yes or no as to whether they were familiar with the rubric. The proportion of teachers who indicated familiarity with the rubric, compared with the radiographer respondents, was minimally larger, with teachers having an SD of 0.192 and radiographers having an SD of 0.393.

Regarding use of the rubric, both groups’ answers ranged from ‘Did not know it existed’ (Response 1) to ‘Used it regularly’ (Response 5), with the most common response being ‘Used it sometimes’ (Response 4).

Table 1 indicates which of the item responses had a significantly different median between the two professional groups.

**Table 1:** Comparison of median responses using the Mann–Whitney test.

	Are medians significantly different? (*P* < 0.05)	Interpretation	*P*-value
Practice experience of educators	No	Similar experience	0.45
Qualifications of the educators	Yes	More radiographers than teachers had certificate or diploma level qualifications rather than degrees	0.04
The educators’ use of the grading rubric	No	Similar use of the grading scale with approximately 80% of both groups using it.	0.54
The rubric was simple to use	Yes	Twenty-one per cent of radiographers disagreed with this statement but only 3% of teachers.	0.0003
The rubric provided an accurate assessment of student performance	Yes	Sixteen per cent of radiographers disagreed with this statement but only 6% of teachers.	0.007
Using the grading scale/rubric has simplified the task of assessing the student's practical performance	Yes	Twenty per cent of radiographers disagreed with this statement but only 7% of teachers.	<0.0001
Using the rubric/grading scale has had a positive effect on student learning	Yes	Twenty-one per cent of radiographers disagreed with this statement but only 8% of teachers.	0.015
The rubric/grading scale has influenced me to think more about assessment	No	Similar responses with around Twenty-one per cent of both groups disagreeing with the statement.	0.195
Do you feel using the grading scale/rubric is more or less reliable than using your judgement to decide on a grade?	Yes	Fifteen per cent of radiographers but only 2% of teachers believed the scale was less reliable than their own judgement.	0.02

The quantitative results indicate that a large majority of the total sample appreciate the value of the grading scale and its capacity to give specific guidance to the assessor for the purpose of simplifying and increasing accuracy when assessing students on professional placement.

### Qualitative

#### Open-ended questions

The qualitative data were analysed using thematic analysis. The open-ended questions in the survey instrument allowed respondents to comment on the best and worst aspects of using the rubric, and these answers followed many different themes. The most commonly recurring themes were the categories as described below. This qualitative data were analysed using NVivo9™ analysis software and was categorised into the following for both teacher and radiographer responses: accuracy of assessment; consistency of assessment; usefulness for feedback to students; use of the rubric compared to personal judgement of the assessor; value for student learning; ease of use; subjectivity of assessment and time factors. Only one category revealed a difference in the median, that of perceived simplicity of using the rubric.

Some specific findings from this analysis are discussed below.

##### Accuracy of assessment

Radiographers’ comments on this theme included those directly or indirectly referring to the effect of rubric use on the accuracy of practical assessment. These were positive, with comments indicating that the rubric facilitated greater accuracy and interrater reliability, and promoted standards-based assessment. This comment is consistent with Boud and Dochy^10^ who stated that ‘…assessment is the making of judgments about how students’ work meets appropriate standards.’ (p. 1).

Among the comments from teacher respondents were those directly or indirectly referring to accuracy. The recurring theme in these comments was that a rubric was a boon to accuracy in practical assessment. The role of the rubric in standards-based assessment was also alluded to, stating that it provided a ‘target’ for students to aim for, and helped teachers to define excellence.

##### Consistency of assessment

Radiographer respondents had little to say about this, with one response stating that the rubric promoted ‘more consistent grading.’ Teachers, however, responded highlighting the importance of consistency, and the positive effect of the rubric in this area. This would appear to reflect the importance that teachers place on consistency of assessment in their school. It is a part of a teacher's daily responsibilities to obtain consistency in the grading of tasks they set for their own students, so it is normal for them to extrapolate this to the pre-service teachers they supervise and assess. The routine practice of radiography, however, does not involve any assessment of student performance, so it is not surprising that there was minimal comment from them in regard to this theme.

##### Usefulness for feedback to students

Radiographer respondents commented positively on this aspect, stating that the rubric allowed students to compare their current level of performance to the level they should be at, thus placing the responsibility for improvement in the hands of the student.

Teacher respondents referred to the usefulness of the rubric for feedback, pointing out that it needed to be ‘used in conjunction with other feedback and assessment strategies’ in order to be truly effective. Other positive responses referred to having a ‘frame of reference’ to be able to align feedback with course objectives. The theme of the rubric being useful in promoting standards-based assessment is seen to again occur under this heading.

##### Use of the rubric compared to personal judgement of the assessor

Radiographer respondents who commented on this category in their answers each had a slightly different slant on it. Comments included the thought that most of the time it was easier to use common sense and compare them to experience with previous students. This answer displays a limited understanding of the role of assessment, and the concept of standards-based assessment. Professional judgement plays an important role in the assessment of competence, whether or not a rubric is used. This is highlighted by Hager et al.^11^ who write ‘Professional judgment plays a crucial role in various aspects of the assessment of competence’ (p. 13) and may be viewed as no less reliable than alternative objective assessment. Comparing students to past experience may not help work towards a uniformly high standard of graduate practitioner.

Another theme was that the rubric provided ‘support and justification’ for allocated grades, which at least demonstrates a desire to have their judgement validated, but raises concerns as to what the assessment might look like if no justification for the supervisor's judgement could be found. The rubric was also seen as being more time consuming than expressing ‘your own thoughts,’ with a qualifying acknowledgement that it is more comprehensive. Teacher respondents commented on the relationship between their judgement and the use of the rubric, and all had similar thoughts. The teachers generally felt that there was no conflict between using the rubric and applying their professional judgement. In fact, they felt that a level of professional judgement was essential in the use of the rubric. An example of their responses in this regard is: ‘Using a grading scale together with judgment is a help in assessing student performance.’

##### Value for student learning

Radiographer respondents commented on the effect of the rubric on the student's learning, with a mixture of positivity and ambivalence. A recurring theme was that it allowed students to identify the areas they needed to improve in. Interestingly, one of the ambivalent comments included the very insightful observation that the rubric ‘needs added space for comments or extra categories for the graders to use as they see fit to allow grading to cover additional features of the students learning.’ This aligns with Sadler,^12^ who introduces the concept of ‘latent’ assessment criteria, which he defines as those ‘in the background, triggered or activated as occasion demands by….some property of the work that deviates from expectation.’ (p. 134).

Teachers had mixed comments about the educational value of the rubric. Sadler's concern about specifying assessment criteria was noted, with comments such as ‘it limited the responses possible.’ Some respondents also commented that they felt individualised feedback was more useful as a learning tool than comparing performance to the rubric. However, others commented that the rubric was useful in identifying what level of expected progress the student had attained, and that it helped students to see what the expectations of them were, and thus assisted the learning process. It would appear that overall the rubric was seen as beneficial to student learning, with some reservations, particularly from teachers.

##### Ease of use

Radiographers agreed that it was simple to apply the rubric to the assessment of their students. Comments included greatly increasing confidence as a first-time student assessor and helping to understand what was required of the student. It would appear that teachers greatly appreciated the rubric's ease of use by making the job of assessing students easier. Particularly, one respondent commented that as a first-time assessor, he would not have been clear about the expectations of the college were without the rubric. The general theme of the responses from both professional groups is that the rubric makes the job of assessing a student's performance easier.

##### Subjectivity of assessment

There was minimal radiographer comment on the subjectivity of student assessment, which suggested that applying the rubric was still affected by subjectivity in the interpretation of the individual criteria. Teachers commented on the subjectivity of using the rubric, but they looked at it from different aspects. Some felt that it removed subjectivity, implying this to be a positive aspect. Others, however, saw removing subjectivity as negative, with subjectivity seen as being a way to distinguish between performances that were close according to the rubric, but significantly different according to the judgement of the supervisor. Responses indicated that the interpretation and application of the rubric's criteria was still quite subjective. These comments are supported by Sadler,^13^ who states that ‘some criteria simply defy expression, even though they are part of the tacit understandings shared by experts’ (p. 68). In other words, ‘professional subjectivity’ is not necessarily a bad thing, as it is using the tacit knowledge developed through experience and reflection to determine the quality of the student's performance.

##### Time factors

The overall impression from radiographers is that they felt that time consumption was a negative aspect of using the rubric, and that this outweighed any positive facets. A suggestion was made that integrating the rubric into the actual assessment tool would make the process ‘easier to follow and less time consuming.’

The opinions of the teachers were divided on the aspect with suggestions that it saved time, that it was time consuming, and even admissions that if they were personally more organised, it would probably save time for them. Another remark was that the rubric contained too many performance indicators and was cumbersome to use, the implication being that using a rubric could be beneficial if the design were simplified.

There is significant common ground in the two disciplines in regard to the attitude of assessors to assessment of students on WIL placement. This focuses on a sense of ‘lack of control’ of tertiary educators over the manner with which placement-based supervisors administer the assessment strategies they are asked to implement.

Given the perennial discussion^7^,^14^ regarding the validity of assessors external to the university giving grades to students rather than a simple pass/fail, the research reported on in this article creates some interesting discussion points. Questions that this article began with still exist but there is now some basis for further discussion. How can there be any cross-placement validity in giving a grade? What are the benefits of a rubric? Do the areas of education and medical imaging differ in the way they consider assessment?

The benefits of students receiving grades for their professional experience are real.^15^ The principle of measuring excellence in performance rather than just pass/fail is pedagogically sound and leads to increased motivation on the part of students to perform well.^2^ Although the rubric helps in the area of consistency and cross-centre validity, the authors of this article accept that this is still an issue.

This research did, however, identify other advantages of using a rubric, including helping supervisors identify key areas in student performance.

It is a reasonable assumption that professional teachers would have a better understanding of the principles of assessment than professional radiographers given that they are trained in education. Radiographers are capable of understanding and applying the principles, but as it is not part of everyday radiography practice, one would not expect a radiographer to have the same experience in their application as a teacher of similar experience, even though they may regularly assess radiography students.

Comments by radiographer respondents such as ‘Revert to a …… system according to the KISS (keep it simple stupid) principle,’ ‘It's not very simple or straightforward;’ and ‘Simplicity is the key’ would indicate that the simpler the system used, the more likely the supervisor is to invest the effort to accurately complete the assessment.

The implications of this study point to the necessity of ease of use and simplicity in an assessment process designed for the WIL environment, without compromising reliability and validity. Clearly, despite a very similar sample for both of the professions in terms of sample size, education and years of experience, there are statistically significant differences in the way teacher supervisors and radiographer supervisors perceive these factors with regard to using a rubric for WIL assessment.

Further research into this area could include comparing the attitudes towards assessment of WIL of a sample of health professionals with postgraduate educational training to those of an otherwise similar sample with no educational training. This would ascertain whether formal educational training is a significant factor in differing attitudes of health professionals.

There were no significant limitations to the study, with good sample sizes, and an excellent response rate from a wide variety of respondents.

## Conclusion

It is indicated by both the quantitative and qualitative data that the majority of respondents from both professions can see the benefits of using a rubric to increase the accuracy and validity of the assessment. For a large number of the respondents the assessment rubric took too much time but many recognised that this extra time was valuable in that it allowed them to accurately reflect the student's performance, without having to guess what the expected standards of performance were. It was also seen to be useful in identifying specific areas for student improvement. Some respondents recognised that the rubric enabled them to justify the allocated grade to the student.

This study has raised more questions while providing some answers. The reliability and validity of student assessment across centres will continue to be an issue, even if all supervisors were to use the grading scale accurately on a regular basis. The supervisors have, however, agreed that using the scale increases the accuracy of the grading. They have also identified several other areas of benefit in its use. These benefits include the focusing of the student and the supervisor on specific skill areas and the empowering of the supervisor to give meaningful feedback to the student. It would have to be concluded that the use of a suitable rubric increases the validity of the assessment process. However, reliability across work placement centres and between supervisors remains an issue.

The data indicate that even though the teacher supervisors are more committed to the use of the rubric, the majority of supervisors in both professions favour its use. However, teachers have a superior understanding of how to use it. On the basis of the results of this study, the authors suggest that education for radiographer supervisors in the principles of sound WIL assessment would increase the meaningfulness of the assessments received. It stands to reason that this can be extrapolated to other health-related professions, who, like radiographers, have clinical supervisors who are experts in their professional fields, but often do not possess the higher education expertise to give a true indication of a student's WIL performance.

## Conflict of Interest

None declared.
